# Knowledge and Perception of Paediatric Drug Dosing: Impact of Paediatric Drug Dosing Workshop

**DOI:** 10.7759/cureus.61140

**Published:** 2024-05-26

**Authors:** Ahmad Kamal Ariffin bin Abdul Jamil, Nur Aqila Amran, Umar Idris Ibrahim, Ng Yen Ping

**Affiliations:** 1 Department of Clinical Pharmacy and Pharmacy Practice, Faculty of Pharmacy, Universiti Sultan Zainal Abidin, Tembila, MYS; 2 Department of Clinical Pharmacy and Pharmacy Practice, Faculty of Pharmacy, Quest International University, Ipoh, MYS

**Keywords:** pharmacy students and workshop intervention, perception, paediatric, knowledge, drug dosing

## Abstract

Background: Paediatric patients frequently encounter medication errors caused by the requirement for individualised drug dose estimates based on weight, age variance, and drug pharmacokinetics. One thing contributing to drug dosing errors is the lack of healthcare personnel's knowledge of paediatric drug dosing. The present study aimed to evaluate the knowledge and perception regarding the workshop on paediatric drug dosing among undergraduate pharmacy students.

Method: A prospective pre-post study was conducted. A virtual workshop on paediatric drug dosing was designed and developed by the clinical pharmacy lecturer from Universiti Sultan Zainal Abidin (UniSZA) for pharmacy students. An online questionnaire with 15 questions regarding knowledge of paediatric drug dosing and perception of the virtual workshop on paediatric drug dosing was used to evaluate pharmacy students’ knowledge pre- and post-workshop.

Result: Twenty-six students took part in the study (100%). In the pre-workshop on paediatric drug dosing calculation, most students had poor knowledge of the paediatric drug dosing calculation, scored 8 out of 15, 26.92% between 9 to 11 and only 11.54% scored ≥ 12. There was a statistically significant difference in median knowledge score between pre- and post-workshop (p< 0.05). Among the students, 73.08% stated that they strongly agreed that the online workshop attracted their attention and 76.92% of students strongly agreed that they were able to calculate paediatric drug dosing after joining the online workshop.

Conclusion: Results demonstrate that pharmacy students have insufficient knowledge of paediatric drug dosing calculations. Virtual workshop is one strategy that could improve the pharmacy students’ knowledge.

## Introduction

Medication errors are a major cause of morbidity and mortality. Children are prone to medication errors because they tend to have high sensitivity to the medications and poor tolerance for errors due to their immature and unique physiological development [1]. The most prevalent form of medication errors among children is dosing errors [2,3]. Furthermore, inappropriate medication dosing has been a leading cause of medication errors [4]. A threefold increase in the risk of adverse outcomes in hospitalised children has been documented compared to the adult population [5]. Children’s weights range from less than 1 kg to more than 100 kg, necessitating numerous dosing regimens [6]. Moreover, multiple calculations also have been involved in determining the medication dose due to the need for weight-based drug dosing which is cited as one of the factors that result in adverse drug events (ADEs) in paediatric patients [5]. Iatrogenic injuries that result in morbidity and mortality have previously been linked to drug dosing calculation errors. Such errors are more likely to be life-threatening in newborns and children [7]. Assuring healthcare professionals can appropriately calculate drug doses is vital in preventing a drug-related adverse event [8]. A pharmacist who is responsible for ensuring the safety and efficacy of medicines that will be consumed by patients, especially children, adequate knowledge of drug dosing calculation should be ensured, as it has been reported that poor knowledge of drug dosing calculation may be one of the factors contributing to inappropriate medicine dosing [1,9]. When choosing dosage, mass, volume, or amount, it is critical to utilise the correct calculating procedures [10]. In surveys of paediatric patients, overdose and low doses are the most commonly reported medication errors. It is evident that incorrect doses are more common than other medication errors [11]. In order to optimise medication therapy, pharmacists must have a thorough understanding of drug doses in paediatric patients. Determining optimal medicine doses in paediatric patients can be difficult for pharmacists who lack knowledge and experience in this area. As a result, undergraduate pharmacy students need to have a strong understanding of paediatric drug doses, as they will be working as practising pharmacists in a hospital or community pharmacy where they will be involved in the medication-use process. A paediatric drug dosing workshop is one option to improve the knowledge of paediatric drug dosing calculations. It has been demonstrated to help enhance mathematical skills in paediatric medicine dosing [12,13].

Therefore, this study aimed to assess the knowledge of paediatric drug dosing among undergraduate pharmacy students and to determine whether there is an improvement in the knowledge level of undergraduate pharmacy students after the virtual workshop on drug dosing in paediatrics. The other objective is to obtain the perception of undergraduate pharmacy students regarding the virtual workshop on paediatric drug dosing.

## Materials and methods

Study design

This prospective pre-post study was conducted in March 2022 among Year 3, Semester 2, Undergraduate Pharmacy (BPharm) students at Universiti Sultan Zainal Abidin (UniSZA) for the academic year 2021/2022.

Study population

The study included all Year 3, Semester 2, Undergraduate Pharmacy (BPharm) students enrolled at UniSZA for the academic year 2021/2022. Inclusion criteria encompassed Year 3, Semester 2, Undergraduate Pharmacy (BPharm) students willing to participate in the research, while exclusion criteria were students who declined to participate and the principal investigator of the study.

Sample size

The sample consisted of 26 students of Year 3, Semester 2, Undergraduate Pharmacy (BPharm) at UniSZA for the academic year 2021/2022. One participant was excluded because he/she was one of the investigators in this study. The class consisted of only 27 students; so 26 represented 96.3% of the study population.

Research tools

Two research tools were employed: an online questionnaire provided via Google Forms and a virtual video concerning paediatric drug dosing calculation. The questionnaire (see Appendices) comprised two sections: Section A collected demographic data (e.g., gender, race, age, Cumulative Grade Point Average (CGPA), while Section B contained 15 multiple-choice questions assessing basic drug dosing calculations in paediatrics, involving 12 different medications. Each correct answer was scored as "1," while incorrect responses were scored as "0," with a total score of 15 marks. The questionnaire's content was adopted from published articles related to drug dosing knowledge and perceptions [14-17]. CGPA is a standardised measurement for varying levels of achievement in the course obtained by a student through his academic journey.

Data collection

The questionnaire was distributed via a Google Forms link. Participants were allotted 40 minutes to complete the questionnaire, during which discussions were not permitted, although they were allowed to use calculators. Access to the questionnaire was restricted after the allotted time. The workshop on paediatric drug dosing was conducted online via Cisco Webex by a clinical pharmacy lecturer from the Faculty of Pharmacy, UniSZA. Two days after the online workshop, the questionnaire was distributed again via a Google Forms link to evaluate the impact of the online workshop. A study by MacDonald et al. (2002) assessed the predictors of forgetting in memory tasks. Comparing the time frame, i.e., 30 minutes, 24 hours, seven weeks, and eight months, accelerated forgetting was significant from 24 hours onwards [18]. This study chose two days as the cut-off time because 24 hours was considered too short to understand the materials taught in the paediatric drug dosing workshop. A time frame of at least one week is too long and can accelerate forgetfulness.

Statistical analysis

Descriptive statistics, including frequency, percentages, and median (interquartile range), were used for data analysis. Bloom's cut-off point was employed to categorize overall knowledge as good, moderate, or poor. The Shapiro-Wilk test assessed data normality, and the Wilcoxon Signed Rank test was used to compare pre- and post-workshop knowledge scores. The significance level was set at p < 0.05.

Ethical considerations

This study received approval from the UniSZA Human Resources Ethics Committee (UniSZA/UHREC/2022/381). Participants' anonymity was maintained. Participants were informed of their right to withdraw from the study at any time. No incentives were offered for participation, and the questionnaire was designed to avoid causing any sensitivities to respondents. 

## Results

Twenty-six (100%) students took part in the study. Nineteen respondents (73.08%) were female and seven respondents (26.92%) were male. Of the 26 students, 88.46% were Malays and 11.54% Indians. More than half of the participants (61.54%) were non-dean list and 38.46% were on the dean list (CGPA ≥ 3.5). Refer to Table 1 for details. 

**Table 1:** Demographic characteristics of the respondents CGPA: cumulative grade point average

Variable		Frequency (N)	Percentage (%)
Gender	Male	7	26.9
	Female	19	73.1
Race	Malays	23	88.46
	Indians	3	11.54
Age (Years)	22	23	88.46
	25	2	7.69
	26	1	3.85
CGPA	Non-dean list	16	61.54
	Dean list (CGPA ≥ 3.5)	10	38.46

Before the virtual workshop on paediatric drug dosing calculation, 61.54% (16) of the students had poor knowledge regarding the paediatric drug dosing calculation (scores < 9), 26.92% (7) scored between 9 to 11 and only 11.54% (3) scored ≥ 12. After the virtual workshop on paediatric drug dosing calculation, 88.46% (23) of the students had good knowledge (score ≥ 12), 7.69% (2) scored between 9 to 11 and 3.85% (1) scored < 9. The median knowledge score of pre-workshop pharmacy students was 8.0 (±2.50) whereas the median knowledge score of post workshop is 13.5 (±2.25). Wilcoxon Signed Rank test was used to compare the knowledge of drug dosing calculation among pharmacy students between pre and post-workshop. The p-value was less than 0.05 (Table 2); thus, there was a statistically significant difference in the median knowledge score between pre- and post-workshop. Refer to Table 2 for details.

**Table 2:** Knowledge score of third-year pharmacy students Wilcoxon Signed Rank test, p < 0.05 = statistically significant IQR: interquartile range

	Pre-workshop (median (IQR))	Post-workshop (median (IQR))	p-value
Knowledge scores among the third-year pharmacy students regarding paediatric drug dosing.	8.0 (2.50)	13.5 (2.25)	0.001

Questions that require knowledge of drug dose

Table 3 represents the calculation questions that require students to know the drug dose either by age or body weight. The frequency and percentage of correct responses are shown in Table 3. Only 26.92% (7) of the students answered question 3 correctly. Most students, 46.20% (12) answered the questions wrongly by choosing a minimum daily dose of the salbutamol instead of the maximum dose. Among the students, 53.85% (14) answered correctly for question 5, which involved the volume per dose of paracetamol suspension in millilitre (mL). Only 38.46% (10) of the students answered question number 7 correctly. For question 13, 69.20% (18) of them to answer the question correctly. For question 14, pharmacy students also showed familiarity with cetirizine dose making the correct response rate of 80.77% (21). Lastly, for question 15, more than half of the pharmacy students answered this question correctly.

**Table 3:** Questions that require knowledge of drug dose either based on age or body weight

Questions (3, 5, 7, 9, 13, 14, 15)	Number of students with correct response (%) in pre-workshop	Number of students with correct response (%) in post-workshop
3. A salbutamol syrup (2 mg/5 mL) was prescribed for a child who weighs 20 kg. What is the maximum safe dose for this child in mL per day?	7 (26.92%)	17 (65.40%)
5. A 7-year-old child (23 kg) was given paracetamol suspension (125 mg/5 mL). Calculate volume in mL per dose if given every 6 hours.	14 (53.85%)	23 (88.50%)
7. A child (8 kg) was given 3 mL per dose of syrup chlorpheniramine (4 mg/5 mL). Calculate the dose in mg that the child got and state whether this is an overdose or not.	10 (38.46%)	24 (92.30%)
9. A child weighed 15 kg taking paracetamol syrup (120 mg/5 mL). What is the maximum safe dose in mL this child can have per day?	10 (38.46%)	20 (76.93%)
13. An azithromycin suspension is supplied in a concentration of 200 mg/ 5 mL. A 2-year-old patient (10 kg) requires the azithromycin for tonsillitis. How much suspension would be given to this patient in mL per day?	18 (69.20%)	23 (88.50%)
14. A 7-year-old male patient has been diagnosed with allergic rhinitis and the doctor prescribed a Zyrtec® cetirizine syrup with strength 1 mg/mL. Which of the following volumes is appropriate for a daily dose?	21 (80.77%)	24 (92.30%)
15. How many mL of Loratadine syrup (5 mg/5 mL) is required for five days for a 7-year-old child?	15 (57.69%)	23 (88.50%)

Questions that require only mathematical skills 

Table 4 represents the calculation questions to assess the mathematical skills of the pharmacy students. The frequency and percentage of correct responses are shown in Table 4. For question 1, 50% (13) of the students gave wrong answers. For question 2, 57.70% (15) of the students gave the wrong answer to this question due to the same condition as described in the first question but the students were able to calculate the dose from mg/kg to mg/mL. On the other hand, for question 4, only 53.85% (14) of the students could answer correctly even though the concepts of the question were similar to the first two questions. Question 6 which involved a mass concentration unit, 65.38% (17) answered the question correctly. Furthermore, in question 8, 69.20% (18) of the students were able to give the correct answer. Refer to Table 4 for details. 

**Table 4:** Questions that do not require the respondents to know the drug dose but only require them to use mathematical skills to solve the problems

Question (1, 2, 4, 6, 8, 10, 11, 12)	Number of students with correct response (%) in pre-workshop	Number of students with correct response (%) in post-workshop
1. Doctor orders Bactrim® suspension (sulfamethoxazole and trimethoprim (TMP)) for a child (10 kg) with a urinary tract infection. The safe dosage range for this medication is 6-12 mg TMP/kg/day every 12 hours. What is the safe dose in mg for this child per dose?	13 (50.00%)	25 (96.15%)
2. Erythromycin ethyl succinate (200 mg/5 mL) is supplied for a 15 kg child with moderate infection. Doctor decided to prescribe a dose of 40 mg/kg/day to be given every 12 hours. What is the volume required per day?	11 (42.30%)	23 (88.46%)
4. Doctor orders amoxycillin suspension for a child weighing 5 kg. The safe dose range for this medication is 45 mg/kg/day divided every 8 hours. What is the maximum safe daily dose in mg for this child?	14 (53.85%)	20 (76.90%)
6. A salbutamol syrup (2 mg/5 mL) is supplied for a child (25 kg) having a bronchospasm. The safe dose range for salbutamol syrup is 0.1-0.15 mg/kg/dose every 6 hours. Which of the following is the minimum volume of salbutamol needed in mL per day?	17 (65.38%)	23 (88.46%)
8. A child requires 10.0 mL Augmentin® suspension (amoxicillin and clavulanic acid) 125 mg/5 mL twice a day for the treatment of acute otitis media. Determine the total dose of Augmentin® suspension in mg per day.	18 (69.23%)	23 (88.46%)
10. Amoxycillin suspension syrup (250 mg/5 mL) is given to a 2-year-old male patient weighing 10 kg with sinusitis. The safe dose range for this medication is 20-40 mg/kg/day in divided doses every 8 hours. Which of the following will be the maximum daily dose in mL for this patient?	19 (73.08%)	24 (92.31%)
11. A 12-year-old patient weighing 30 kg requires 10 mg/kg/dose twice daily of cefuroxime suspension (125 mg/5 mL). How much suspension would you supply per dose to the patient?	21 (80.77%)	25 (96.15%)
12. An 8-year-old child with a weight of 12 kg requires 5 mL bromhexine syrup (4 mg/5 mL) three times daily. How much bromhexine will the patient receive each day in mg?	21 (80.77%)	25 (96.15%)

## Discussion

A significant concern regarding medication dose calculation competency and the risk to patient safety has been documented in previous studies [19,20]. It is vital to ensure that pharmacy students have adequate knowledge about the drug dosing calculations to ensure the patient’s safety. For overall individual scores, most pharmacy students were categorised as having inadequate knowledge regarding paediatric drug dosing calculations. Only 11.54% (3) of the students answered more than 80% of the questions correctly. Furthermore, for the questions involving the knowledge of drug dosing, more than half of the students answered correctly for each question, including paracetamol, cetirizine, loratadine, and azithromycin (Table 3). This indicates that pharmacy students knew the paediatric dose for paracetamol, cetirizine, loratadine and azithromycin. The four-answer options in multiple-choice questions format (see Appendices) allowed a 25% chance that the students would select the correct answer. Furthermore, the previous studies also reported that students shared that having the choice of answer written out in a list gave them more confidence in their calculations, which might affect overall performance [19,21]. The results of question 3 showed that the students had inadequate knowledge regarding the children’s dose range of salbutamol syrup. Previous studies reported that salbutamol is a drug commonly associated with dosing errors [22,23]. This could be due to the lack of knowledge regarding the dosing of salbutamol in paediatric patients. For question 5, this could be due to familiarity with paracetamol, which is one of the most commonly used antipyretics, especially for paediatrics [24]. For question 7, most of the students showed capability in calculating doses based on the given volume but were unable to determine whether the dose was overdosed or not due to their lack of knowledge of chlorpheniramine dose per body weight. Question 9 showed a lower score compared to question 5 due to the changes in the unit of the required dose. The previous question acquired dose in mL/dose whereas this question is in mL/day. 

Most of the students answered the question in mL/dose, assuming that the students might either not be concerned about the unit or were not fully aware of the dose frequency. The dosing unit is vital to ensure the correct calculation. As for question 13, it aligns with the findings of Keewan et al., who also discovered that a majority of participants correctly answered the question pertaining to the dosing of azithromycin [9]. This is because the azithromycin dose for tonsilitis does not involve the range dose, making it easier for memorisation. The question in the study by Keewan et al. also used a similar dose as in the present study [9]. For question 14, the percentage of correct answers is high because the dose was indicated by the child’s age instead of the child’s body weight is easier. The study by Özyazıcıoğlu et al. stated that a question about calculating doses depending on age is the most prevalent way in paediatrics and gives assurance in determining the correct dose [11]. More than half of the respondents got a correct answer for question 15 due to the easier dose of loratadine which is also based on the child’s age similar to cetirizine.

As for questions in Table 4, half of the students gave wrong answers to question 1 which could be due to their carelessness in calculating the drug dose without taking into consideration the expression of dose units required for the calculation (mg/dose) and the unit given in the questions (mg/day). For question 2, this could be due to the students preferring the dose calculation in mass concentration, which has also been reported in the previous study [8]. Thus, this proved that the students had poor knowledge regarding the dose of salbutamol in paediatric as most of them could answer question 6 correctly while for question 3 most of them answered wrongly once the dose was not given in the question. For question 8, in contrast to the study by Keewan et al., most of the students answered wrongly the question that involved Augmentin® dose calculation [9]. This could be due to the question design, which differs from the current study. The results of question 10 suggest that students who were more interested in determining the dose in mL instead of mg, thus influencing them to make thorough calculations and preventing them from carelessness in calculations related to the dose unit. As for the result for question 11, the results are inconsistent with a previous study which stated that the students only performed fairly in dose calculation of cefuroxime suspension [25]. As for question 12, it could be concluded that dose unit expressions did not necessarily influence the students to calculate the dose correctly but they may have been rushing to answer the questions due to the time limit set to complete the questionnaire. Thus, the present study showed that the pharmacy students had considerable skills in mathematical calculations but carelessness in a given unit of drug dose led them to answer wrongly. Previous studies reported that students performed poorly in the calculations of drug dosing because they lacked numeracy skills and did not reveal the carelessness of the students regarding the dose unit required by the questions that led the students to make mistakes in the calculation of the drug dose [19,26].

Before the virtual workshop, students had difficulty performing paediatric drug dosing calculations, especially in questions involving the knowledge of drug dosing. OF the students, 61.54% (16) obtained a score of less than 9 with a median score of 8. This is similar to the study by Devi et al. which revealed that the pharmacy students’ ability in paediatric drug dosing calculation was lacking before the intervention [27]. Besides, half of the students (eight students) with poor knowledge were able to score at least 8 points in the pre-workshop. This finding showed that pharmacy students might have considerable competency in mathematical skills. After the workshop, the current study revealed a significant improvement in pharmacy students’ knowledge. In the post-workshop calculations test, the majority of the students (n = 23) performed well, showing a significant impact of the workshop. The knowledge of pharmacy students regarding the paediatric drug dosing calculations increased significantly. This finding is similar to studies, which also reported the effectiveness of workshop intervention in improving pharmacy students’ knowledge [12, 13]. 

Thus, overall findings suggest that an online workshop is an effective way to improve pharmacy students’ knowledge of paediatric drug dosing calculations. 

This study has a few limitations, firstly it considers data from one pharmacy school in Malaysia; it is not necessarily representative of student perceptions and knowledge in other institutions, either in Malaysia or internationally. Additionally, as this study was conducted online, the possibility that the students might be discussing the questions could not be avoided thus this could have influenced the results obtained. There is also the potential for bias because the participants are among the researcher’s peers. Therefore, the response may overestimate the perception of the virtual workshop. Lastly, the four-answer option multiple-choice question format allows at least, a 25% chance of blindingly choosing the answer correctly.

## Conclusions

This study illustrated that pharmacy students have poor knowledge of paediatric drug dosing and inadequate mathematical skills in performing drug dosing calculations. Implementing interventions that can help pharmacy students improve their knowledge is vital as they will become a pharmacists. This study demonstrated that an online workshop significantly impacted pharmacy students’ knowledge of paediatric drug dosing. Thus, an online workshop can be utilised as one of the learning methods to improve the knowledge of paediatric drug dosing among pharmacy students and other healthcare students.

## Appendices

Questionnaire

Section A: Demographic Information 

1)    Gender 

              Male

              Female

2)    Race

              Malay

              Chinese                         

              Indian

              Others

3)    Age:  

4)    Grade point average (CGPA): 

Section B: Calculation for Drug Dosing in Paediatrics

1.     Doctor orders Bactrim® suspension (sulfamethoxazole and trimethoprim (TMP)) for a child (10 kg) with a urinary tract infection. The safe dosage range for this medication is 6-12 mg TMP/kg/day every 12 hours. What is the safe dose in mg for this child per dose?

a)    120 mg/dose

b)    30 mg/dose 

c)    110 mg/dose

d)    100 mg/dose

2.     Erythromycin ethyl succinate (200 mg/5 mL) is supplied for a 15 kg child with moderate infection. Doctor decided to prescribe a dose of 40 mg/kg/day to be given every 12 hours. What is the volume required per day?

a)    15 mL

b)    7.5 mL

c)    30 mL

d)    3.3 mL

3.     What is the maximum safe dose for this child in mL per day? A salbutamol syrup (2 mg/5 mL) was prescribed for a child who weighs 20 kg. What is the maximum safe dose for this child in mL per day?

a)    30.0 mL

b)    7.5 mL

c)    2.0 mL

d)    20.0 mL

4.     Doctor orders amoxycillin suspension for a child weighing 5 kg. The safe dose range for this medication is 45 mg/kg/day divided every 8 hours. What is the maximum safe daily dose in mg for this child?

a)     675 mg

b)     28 mg

c)     75 mg

d)     225 mg 

5.     A 7-year-old child (23 kg) was given paracetamol suspension (125 mg/5 mL). Calculate volume in mL per dose if given every 6 hours.

a)    9.2 mL/dose

b)    13.8 mL/dose 

c)    55.2 mL/dose

d)    36.8 mL/dose

6.     A salbutamol syrup (2 mg/5 mL) is supplied for a child (25 kg) having a bronchospasm. The safe dose range for salbutamol syrup is 0.1-0.15 mg/kg/dose every 6 hours. Which of the following is the minimum volume of salbutamol needed in mL per day?

a)    37.5 mL/day

b)    25 mL/day 

c)    6.25 mL/day

d)    9.38 mL/day

7.     A child (8 kg) was given 3 mL per dose of syrup chlorpheniramine (4 mg/5 mL). Calculate the dose in mg that the child got and state whether this is an overdose or not.

a)    2.40 mg, not overdose

b)    2.40 mg, overdose 

c)    3.75 mg, not overdose

d)    3.75 mg, overdose

8.     A child requires 10.0 mL Augmentin® suspension (amoxicillin and clavulanic acid) 125 mg/5 mL twice a day for the treatment of acute otitis media. Determine the total dose of Augmentin® suspension in mg per day.

a)    500 mg/day 

b)    250 mg/day

c)    62.5 mg/day

d)    31.35 mg/day

9.     A child weighed 15 kg taking paracetamol syrup (120 mg/5 mL). What is the maximum safe dose in mL this child can have per day?

a)    37.50 mL 

b)    9.38 mL

c)    14.38 mL

d)    2.67 mL

10.  Amoxycillin suspension syrup (250 mg/5 mL) is given to a 2-year-old male patient weighing 10 kg with sinusitis. The safe dose range for this medication is 20-40 mg/kg/day in divided doses every 8 hours. Which of the following will be the maximum daily dose in mL for this patient?

a)    2.7 mL/day

b)    4.0 mL/day

c)    8.0 mL/day 

d)    3.1 mL/day

11.  A 12-year-old patient weighing 30 kg requires 10 mg/kg/dose twice daily of cefuroxime suspension (125 mg/5 mL). How much suspension would you supply per dose to the patient?

a)    12 mL/dose 

b)    24 mL/dose 

c)    1.0 mL/dose

d)    2.1 mL/dose

12.  An 8-year-old child with a weight of 12 kg requires 5 mL bromhexine syrup (4 mg/5 mL) three times daily. How much bromhexine will the patient receive each day in mg?

a)    15 mg 

b)    12 mg 

c)    20 mg

d)    18.75 mg

13.  An azithromycin suspension is supplied in a concentration of 200 mg/ 5 mL. A 2-year-old patient (10 kg) requires the azithromycin for tonsillitis. How much suspension would be given to this patient in mL per day?

a)    3.0 mL 

b)    0.6 mL

c)    8.3 mL 

d)    12.5 mL

14.  A 7-year-old male patient has been diagnosed with allergic rhinitis and the doctor prescribed a Zyrtec® cetirizine syrup with strength 1 mg/mL. Which of the following volumes is appropriate for a daily dose?

a)    2.5 mL

b)    12 mL

c)    5 mL 

d)    3.5 mL

15.  How many mL of Loratadine syrup (5 mg/5 mL) is required for five days for a 7-year-old child?

a)    25 mL 

b)    50 mL

c)    12.5 mL 

d)    35 mL
